# Can the Contents of Biogenic Amines in Olomoucké Tvarůžky Cheeses Be Risky for Consumers?

**DOI:** 10.3390/foods14030456

**Published:** 2025-01-31

**Authors:** Eva Samková, Eva Dadáková, Kateřina Matějková, Lucie Hasoňová, Simona Janoušek Honesová

**Affiliations:** 1 Department of Food Biotechnologies and Agricultural Products’ Quality, Faculty of Agriculture and Technology, University of South Bohemia in České Budějovice, Studentská 1668, 370 05 České Budějovice, Czech Republic; samkova@fzt.jcu.cz (E.S.); maliks00@fzt.jcu.cz (S.J.H.); 2 Department of Applied Chemistry, Faculty of Agriculture and Technology, University of South Bohemia in České Budějovice, Studentská 1668, 370 05 České Budějovice, Czech Republic; dadakova@fzt.jcu.cz (E.D.); matejkova@fzt.jcu.cz (K.M.)

**Keywords:** smear-ripened cheeses, biogenic amines, polyamines, batch, shape, storage, active acidity, salt content

## Abstract

Smear-ripened cheeses are fermented dairy products characterised by an increased content of biogenic amines (BAs). The high contents of these bioactive compounds can negatively affect consumers. The study aimed to observe the contents of BAs and po-lyamines (PAs) in Olomoucké tvarůžky cheeses depending on selected factors (year, batch, ripening/storage time, shape, weight, specific surface area, acidity, and salt content). The results showed that the variability was explained primarily by the batch (83% for the sum of BAs) and by the year (63% for the sum of PAs). The storage time significantly influenced the contents of putrescine, cadaverine, spermidine, and spermine (the explained variability was only 1–3%). The total BA contents negatively correlated with weight (r = −0.6374; *p* < 0.001) and positively with specific surface area (r = +0.4349; *p* < 0.001). A negligible positive correlation coefficient was found between the total BAs and pH (r = +0.1303). A low negative correlation was also found between the total BAs and salt content (r = −0.1328). Compared to previous studies, the total average BA contents were considerably low. In conclusion, this type of cheese does not represent a serious problem for most consumers.

## 1. Introduction

Biogenic amines (BAs) and polyamines (PAs) are small organic compounds with at least one amine group. They have an alkaline character and can be bonded to acidic components in various biological materials. The BAs and PAs are classified by their chemical structure (aliphatic, aromatic, heterocyclic) or a number of amine groups (monoamine, diamines, polyamines). They are very intensively studied because of their biological activity (e.g., [1,2,3]).

Both groups of so-called natural amines have different mechanisms of origin. PAs (putrescine—PUT, spermine—SPM, and spermidine—SPD) are associated with the metabolism of arginine or ornithine [4]. These compounds have an essential role in cell division and tissue regeneration. Further, they have cardioprotective effects and help maintain homeostasis. Their presence in all living organisms is normal and desirable [2,5,6]. The biosynthesis of PAs is attenuated with age; thus, the importance of exogenic sources increases. The BAs (tryptamine—TRM, tyramine—TYM, 2-phenylethylamine—PEA, cadaverine—CAD, and histamine—HIM) arise mainly through the decarboxylation of free amino acids. For example, HIM is formed from histidine and TYM from tyrosine [7,8]. Some BAs can also originate from a two-step reaction (decarboxylation and deamination) or the amination and transamination of aldehydes or ketones during metabolic processes [9].

The main reasons for monitoring the amine contents are their usefulness as an indicator of food degradation and their potential toxicity for consumers [3]. BAs, especially HIM and TYM, have many physiological and toxicological effects, and their high consumption can cause health problems [1,10,11].

Ripened cheeses are among the foods that meet all the conditions necessary for the formation of BAs, i.e., high protein content and decarboxylase-active microorganisms [8,12]. Surface mould-ripened (e.g., Camembert), blue-veined (e.g., Roquefort), and smear-ripened cheeses (e.g., Limburger) are considered the most problematic in this regard [13,14,15,16,17,18,19].

In particular, high amounts of TYM (>100 mg·kg^−1^) can cause symptoms of food poisoning, such as headaches, migraines, nausea, and weakness. TYM is most dangerous for people who are treated with monoamine oxidase inhibitors (MAOI), e.g., antidepressants. In them, TYM, even in small amounts, can lead to a so-called cheese reaction associated with a life-threatening increase in blood pressure. Therefore, these people should avoid foods containing high TYM, including aged cheeses [11,20,21]. On the other hand, these cheeses contain many aromatic compounds due to the profound disintegration of basic components, which causes characteristic sensory properties [22,23] favoured by many consumers.

Olomoucké tvarůžky (OT; Olomouc curd cheese) is the original Czech smear-ripened cheese with a PGI (Protected Geographical Indication) designation [24] produced from curd obtained through acid coagulation (without rennet) of pasteurised cow milk. The processing technology of OT is briefly described by Komprda et al. [25]. During ripening, OT acquires a unique spicy taste and typical flavour, a surface covered with a golden-yellow smear caused by *Brevibacterium linens*, and a cohesive soft consistency with a lighter core. Fat content does not exceed 1%, and the dry matter content is at least 33%. The shapes are generally discs, rings, cylinders, or irregular pieces [26].

OT is one of the most popular cheeses among Czech consumers because of its distinctive characteristics. Given the risks mentioned above, assessing the OT’s BA content, including the affecting factors, is essential. Therefore, the aim of the study was to evaluate the contents of the main BAs (PUT, CAD, HIM, TYM) and PAs (SPD and SPM) in OT samples depending on the year, batch/season, the ripening/storage time, and the shape of the cheese in the long-term period. The study also assessed the relationships between BA contents and selected OT characteristics.

## 2. Materials and Methods

### 2.1. Cheese Sampling

OT samples of different shapes (discs, cylinders, and rings) from one manufacturer (A.W. spol.s r.o., Loštice, Czech Republic) were obtained in the retail network in three years (2011, 2013, and 2019). Within each of the 17 samplings, 5 samples of each available shape were purchased. The entire package of cheese was always used for the analyses. According to the manufacturer, the average nutritional value per 100 g is as follows: energy: 541 kJ/127 kcal; fat: 0.5 g, of which saturated fatty acids: 0.3 g; carbohydrates: 2.6 g, of which sugars: ˂0.4 g; protein: 28 g; and salt: 4.5 g [26].

Samples were obtained approximately two weeks after their production. They were stored at 5 ± 0.5 °C for four weeks in the laboratory and analysed at the following times: (i) two weeks (2WB) before the minimum durability date (MDD), i.e., best before date, (ii) one week (1WB) before the MDD, (iii) at the MDD (MDD), and (iv) one week (1WA) after the MDD and (v) two weeks (2WA) after the MDD. In total, 85 samples were analysed (Table 1).

### 2.2. Chemical Analysis

#### 2.2.1. Chemicals and Standards

Putrescine dihydrochloride 99%, cadaverine dihydrochloride 99%, histamine dihydrochloride 99%, tyramine hydrochloride 97%, and 1,7-heptanediamine 97% (internal standard) were purchased from Fluka AG (Buchs, Switzerland). Spermidine trihydrochloride 99.5%, spermine tetrahydrochloride 99.5%, and dansyl chloride 95% were purchased from Sigma–Aldrich, St. Louis, MO, USA. Other chemicals: proline, heptane (Fluka AG, Buchs, Switzerland); acetonitrile (Merck KGaA, Darmstadt, Germany); Na_2_CO_3_, K_2_CO_3_, AgNO_3_, and acetone (Penta s.r.o., Chrudim, Czech Republic); NaHCO_3_ (Lach-Ner s.r.o., Neratovice, Czech Republic); and perchloric acid (Acros Organics BV, Geel, Belgium), buffer solutions pH 4 and 7 (Hamilton, Bonaduz AG, Switzerland). All chemicals were of analytical grade or higher. Deionised water was prepared with Premier equipment (Premier Systems, Phoenix, AZ, USA). A standard (stock) solution of BAs was prepared at a concentration of around 400 mg/L in 0.6 M HClO_4_ and was further diluted for experiments.

#### 2.2.2. Determination of BA and PA

##### Sample Extraction for Amine Analysis

The food sample (40 g) was homogenised in a plastic beaker with 100 mL of 0.6 M HClO_4_ for 3 min. The mixture was centrifuged for 10 min at 1800 g with centrifuge Sigma 2-5 Centrifuge (Sigma Laborzentrifugen GmbH, Osterode am Harz, Germany). The supernatant was filtered through a filter paper (Filpap, Štětí, Czech Republic), and the volume was recorded. One millilitre of the acidic extract was spiked with 100 μL of internal standard solution (1,7-heptanediamine, 400 mg/L) and placed in a plastic test tube with 1.5 mL of carbonate buffer pH 11 [27]. After vortexing, 2 mL of dansyl chloride solution was added (dansyl chloride in acetone 5 mg/mL) as a derivatisation agent. The test tube was shaken at room temperature for 20 h in darkness. Subsequently, 200 µL of proline solution (100 mg/mL) was added, and the sample was shaken for an additional 1 h. The dansyl derivatives were extracted with 3 mL of heptane. One millilitre of the extract was dried at 60 °C under a stream of nitrogen (Thermovap Ecom s.r.o., Prague, Czech Republic). The dry residue was dissolved in 1.5 mL of acetonitrile. Samples were filtered through glass fibre filters (1.7 μm, Filpap, Štětí, Czech Republic) using filtration assembly Swinnex (Millipore, Carrigtwohill, Ireland) before analysis.

##### The Chromatographic Conditions

Dansyl derivatives were separated using a 1200 Series Rapid Resolution LC System (Agilent Technologies, Inc., Santa Clara, CA, USA). The system was equipped with binary pumps, a micro-vacuum degasser, a high-performance autosampler, and a diode array detector. Column Zorbax Eclipse XDB C-18 (4.6 × 50 mm, particle size 1.8 µm; Agilent Technologies, USA) was used. Chromatographic separation was carried out using a gradient elution of (A) acetonitrile (100%), (B) acetonitrile (50%) as follows: 0–2 min, A 40%, B 60%, 2–3 min, A 40–80%, B 60–20%, 3–4 min, A 80–90%, B 20–10%, 4–6 min, A 90–95%, B 10–5%, 6–7 min, A 95–40%, B 5–60%, 7–12 min, A 40%, and B 60%. Linear concentration change was performed in all cases. Analytical data were evaluated using ChemStation for LC 3D systems (Agilent Technologies, Santa Clara, CA, USA).

##### Calculation and Method Parameters

The evaluation was carried out according to Moschou et al. [4]. The chromatographic data were processed using the program ChemStation for LC 3D systems (Agilent Technologies, Inc., Santa Clara, CA, USA). Calibration and basic calculations were carried out using MS Office Excel.

The parameters of the analytical method used are given in Table 2.

#### 2.2.3. Determination of Physical–Chemical Properties

##### Surface and Specific Surface Area Calculation

The dimensions for calculation of surface area and specific surface area (radius, height, weight) were measured with an accuracy of 1 mm.

The surface area (mm^2^) of the cheese was calculated as the surface area of a cylinder for disc cheese (S_1_) or a cylinder with a central hole for ring cheese (S_2_). The specific surface area (mm^2^/g) represents the ratio of surface area to the weight of a given cheese (Table 1).

S_1_ = 2 × π × r^2^ + 2 × π × r × h, (1)

S_2_ = 2 × π × (r_1_^2^ − r_2_^2^) + 2 × π × h × (r_1_ + r_2_), (2)

where S_1_ = the surface area (mm^2^) of a cylinder for disc cheese, S_2_ = the surface area (mm^2^) of a cylinder with a central hole for ring cheese, r = radius (mm), h = height (mm), r_1_ = outer radius (mm), and r_2_ = inner radius (mm).

##### Determination of pH Value in Cheeses

Determination of pH value was made according to Cvak et al. [28]. A combined puncture pH electrode HC 123 (Fisher Scientific, Pardubice, Czech Republic) with pH-meter pH 700 (Eutech Instruments, Singapore) was used to determine the pH value in cheeses. The electrode used was calibrated using two standard pH 4 and 7 solutions. Before measurement, the electrode was calibrated using two standard solutions of pH 4 and 7.

##### Determination of NaCl Content

The content of NaCl was determined in the aqueous extract by direct argentometric titration, according to Cvak et al. [28]. The weighted amount of cheese (10 ± 0.01 g) was homogenised in 25 mL of warm distilled water using a laboratory homogeniser (Ika Ultra-Turrax, Merck KGaA, Darmstadt, Germany). The mixture was centrifuged (Sigma Laborzentrifugen GmbH, Osterode am Harz, Germany) at 3500 rpm for 4 min (1800× *g*). The supernatant was filtered through a paper filter and topped up to the exact volume in a 250 mL volumetric flask. An amount of 25 mL of the filtrate was titrated with a volumetric solution of silver nitrate, and titration was performed in triplicate.

### 2.3. Statistical Analysis

Statistica 12.0 software (StatSoft CR s.r.o., Prague, Czech Republic) was used for statistical evaluation (descriptive statistics, a general linear model, two-way ANOVA, and correlation analysis). The assumption of using parametric methods (normality of the data and homogeneity of variances) was made for the dataset.

A general linear model with the fixed effect of year and ripening/storage period and with the effect of batch (sampling of different shapes) nested in the year was used:

Y_ijkl_ = μ + Y_i_ + B_j_ (Y_i_) + P_k_ + ε_ijkl_, (3)

where Y_ijkl_ = content (mg/kg) of PUT, CAD, HIM, TYM, SPD, SPM, BA4 (sum of PUT, CAD, HIM, and TYM), and PA2 (sum of SPD and SPM); μ = mean; Y_i_ = year (i = 3; 2011, 2013, and 2019); B_j_ (Y_i_) = batch of shapes (j = 17; six samplings of rings, four samplings of small discs, three samplings of large discs, and four samplings of cylinders); P_k_ = period (k = 5; 2WB, 1WB, MDD, 1WB, and 2WB); and ε_ijkl_ = residual error.

Tukey’s HSD test was used for group comparisons (post hoc tests). The total explained variance (coefficient of determination; R^2^) and variance explained by year, batch, and ripening/storage time (factors variance) were calculated using the sum of squares and expressed in %. R^2^ was defined as [(1 − (residual sum of squares/total sum of squares)) × 100], and factors’ variance was defined as [(sum of squares of individual effects/total sum of squares) × 100].

A two-way ANOVA with the fixed effect of shape, storage, and interaction (shape × ripening/storage period) was used:

Y_ijk_ = μ + S_i_ + Pj + (S × P)_ij_ + ε_ijk_, (4)

where Y_ijk_ = content (mg/kg) of CAD, SPM, and BA4 (sum of PUT, CAD, HIM, and TYM); μ = mean; S_i_ = shape (i = 4; rings, cylinders, small discs, and large discs); P_j_ = period (k = 5; 2WB, 1WB, MDD, 1WB, and 2WB); and ε_ijk_ = residual error. Tukey’s unequal N HSD test was used for group comparisons (post hoc tests).

Pearson correlation coefficients (r) were used at the usual levels of significance (0.05, 0.01, 0.001).

## 3. Results and Discussion

### 3.1. The Contents of BAs and PAs in OT Samples Depending on Batch, Year, and Storage Time

The production of smear-ripened cheeses has a long tradition in Europe [29]. Well-known ones include Tilsit, Romadour, Limburger, Harzer, Münster, and Weinkäse [30]. Due to their attractive sensory properties, these cheeses are widely popular among consumers. Although the available statistics do not provide any information on the consumption of these cheeses, recent estimates indicate that smear-ripened cheeses account for 5–15% of the European cheese market [29].

However, smear-ripened cheeses are also associated with a risk of BAs, and therefore, it is important to investigate their levels, which also applies to OT. Our study investigated contents of BA and PA in OT depending on year, batch, and ripening/storage time. The batch was characterised by the cheese shape, month, and year. 

In the total of 17 samplings, each representing one batch, considerable differences were found in the average contents of both individual BAs and PAs and their groups (BA4 and PA2)—Table 3. This is most evident in the contents of CAD. The lowest CAD content (1.66 mg/kg) was determined in Rings 3 (March/2013), and the highest (128 mg/kg) in Discs–small 1 (October/2011). On the other hand, small ranges were found for PA2 (3.38–16.1 mg/kg). 

In most tested batches (65%), TYM was among the BAs with the highest proportion. These results are consistent with the literature, e.g., [11,15,25]. 

The highest average contents of health-important BAs, i.e., HIM and TYM, were recorded in Cylinders 3 (May/2013) (57.1 mg/kg) and Discs–small 2 (March/2013) (64.6 mg/kg), respectively. In most cases, the sum of HIM and TYM was below 100 mg/kg. The only exception was Cylinders 3 (May/2013), where the sum was 114.7 mg/kg (the sum of the average contents of HIM and TYM given in Table 3).

The European Food Safety Authority (EFSA) [31] states 50 mg HIM per serving as the limit value for healthy people. However, in the case of proven histamine intolerance, only zero HIM (below the limit of quantification) is tolerated. For cheese, a dose of 50 mg of HIM per serving is also recommended as safe [32]. A dose of 6 mg of TYM can cause hypertensive symptoms during classical (first-generation) MAOI treatments [31,33], whereas up to 50–100 mg of TYM can be tolerated by persons treated with third-generation MAOI drugs [34]. According to McCabe-Sellers et al. [33], a dose of 6–10 mg of TYM is associated with a mild reaction and 10–25 mg of TYM with a severe reaction in a person who is taking classical MAOI drugs. Healthy people can digest 200–800 mg of TYM before experiencing increased blood pressure. 

In our study, the TYM content varied considerably between the batches, from 3.17 mg/kg (Discs–large 3; May/2013) to 64.6 mg/kg (Discs–small 2; March/2013). This indicates that sensitive people cannot rely on one so-called “good” experience. Considering the highest average TYM content in our study (i.e., 64.6 mg/kg), 774 and 1548 g of OT would be required to achieve 50 and 100 mg TYM, respectively. This is, of course, a hypothetical consideration. On the other hand, the lowest hazardous dose of TYM (i.e., 6 mg) could be easily reached by consuming 93 g of OT. When assessing the risk of TYM, it should also be taken into account that cheeses are not the only source of BAs. Other foods and beverages typically consumed with cheeses may also contain high levels of BAs, e.g., fermented meat products, wines, and beer [11,12].

Furthermore, BAs should be considered together as there is the possibility of synergistic interactions [31,33]. In our study, the total sum of four BAs (BA4) exceeded a value of 100 mg/kg in five batches (29%), of which only one was 242 mg/kg (Discs–small 1 (October/2011)).

In addition, there are also various factors altering the detoxification of dietary BAs and allowing for the development of adverse effects, such as smoking [35] and alcohol consumption [36]. Individual susceptibility to BAs must also be considered [3].

BA contents in OT have been investigated by relatively few authors so far. These authors focused mainly on the effect of storage conditions, i.e., time and temperature [15,25]. The evaluation of different shapes is rare. For example, Komprda et al. [25] mentioned a comparison of small discs and bars. The authors found significantly higher contents of TYM and HIM in the discs than in the bars and no significant differences in CAD contents. These data are consistent with our findings. 

The contents of BAs depend on various factors. Previous studies have confirmed that the BA contents vary depending on milk quality, microbial starter culture, the presence of microorganisms producing decarboxylases, the type of cheese, technology processing, ripening/storage conditions, etc. [15,25,37,38].

Our study’s general linear model showed a high proportion of total explained variability for all BAs and PAs. It can be seen in the coefficients of determination (R^2^), which ranged from 78.0% (TYM) to 97.4% (HIM). The high total explained variability was mostly caused by the batch and the year. However, the proportions of these two factors differed enormously depending on the groups BA4 and PA2. The highest proportions of explained variability were the batch (83%) for BA4 and the year (63%) for PA2. This phenomenon may be related to the different formation of individual BAs and PAs. While PAs are more associated with the raw material, i.e., milk and its quality, BAs are related to the technological process and cheese ripening.

OT belong to smear-ripened cheeses, the typical sensory properties developing gradually with storage time. Our study was designed to capture the changes in the content of dominant BAs and PAs of OT samples before and after the MDD.

**Figure 1:**
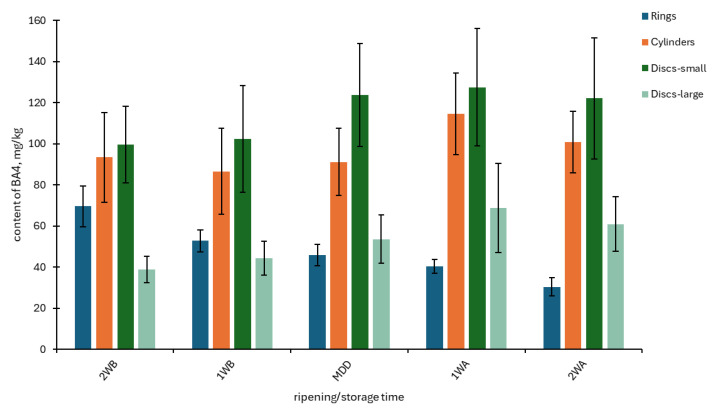
Total sum of four BAs (putrescine + cadaverine + histamine + tyramine) monitored during 28 days of storage in four shapes of Olomoucké tvarůžky cheeses. 2WB = two weeks before minimum durability date (MDD); 1WB = one week before MDD; MDD = date of MDD; 1WA = one week after MDD; 2WA = two weeks after MDD.

The content of the BA4 group increased during the ripening/storage time in all cheese shapes except for Rings (Figure 1). The BA4 content increased mainly in Discs–large, from 38.9 mg/kg to 60.9 mg/kg, which is 156% of the initial content; the increase was lower in other shapes. The content of the evaluated BA4 in Rings decreased from 69.7 mg/kg to 30.5 mg/kg, i.e., to 43.7% of the initial content. A decrease in BA content during long-term storage was also recorded in other studies [39,40]. However, BAs were generally low, and changes were not always statistically significant, as demonstrated in our previous study [13].

**Figure 2:**
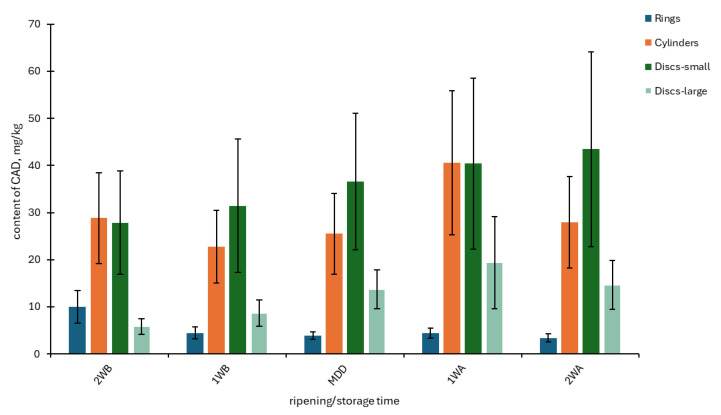
Content of cadaverine (CAD) monitored during 28 days of storage in four shapes of Olomoucké tvarůžky cheeses. 2WB = two weeks before minimum durability date (MDD); 1WB = one week before MDD; MDD = date of MDD; 1WA = one week after MDD; 2WA = two weeks after MDD.

**Figure 3:**
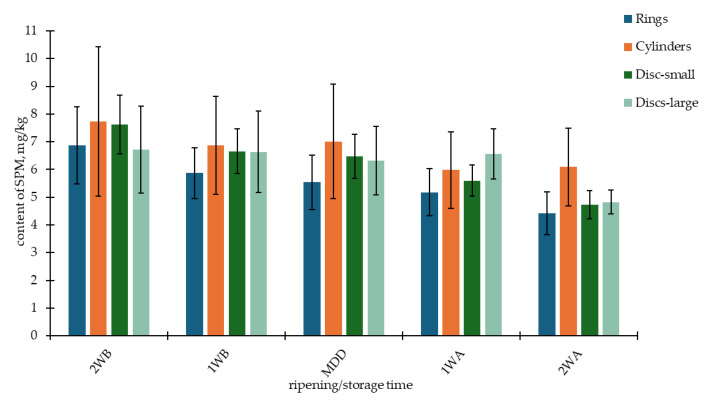
Content of spermine (SPM) monitored during 28 days of storage in four shapes of Olomoucké tvarůžky cheeses. 2WB = two weeks before minimum durability date (MDD); 1WB = one week before MDD; MDD = date of MDD; 1WA = one week after MDD; 2WA = two weeks after MDD.

The factor of ripening/storage time had a statistically significant effect only on the contents of PUT, CAD, SPD, and SPM (see Table 3). Still, considering the previous factors (batch and year), this proportion was minimal (<0.5–5%). A different trend is evident for CAD (an increase in content) (Figure 2) and SPM (a decrease in content) (Figure 3). The content of polyamines SPM and SPD decreased during storage. These amines are important for cell growth and division; therefore, their content may decrease during microbial activity. This trend was also observed during the ripening of mould cheeses [41].

However, these results do not entirely correspond with the results of other authors who noted a statistically significant increase in BA contents in OT during storage [15,25]. In those studies, the maximum rise in BA contents was observed only after an extended storage time (up to seven weeks). A statistically significant increase in BA contents during storage was also observed in blue-veined cheeses [14]. The storage temperature used was also different among the studies. In our experiment, OT samples were stored at 5 °C for 28 days, while Komprda et al. [25] used two storage temperatures (5 °C and 20 °C) for 43 and 66 days. In our work, the total time from the production of OT to the end of the experiment was approximately 42 days.

Overall, it can be concluded that even with extended storage, risky BA (BA4) content did not increase the above levels, which could cause problems in healthy people.

### 3.2. The Relationships Between BA Contents and Selected Chemical–Physical Properties of OT Samples

The differences in BA contents of various OT shapes, mentioned in Section 3.1, could also be related to other characteristics of OT. It is known that the BA contents on the surface/edge are higher than those inside/core of cheeses. These results were confirmed, for example, by Komprda et al. [42] for Dutch-type cheeses, Standarová et al. [14] for blue-veined cheese Niva, and Marijan et al. [43] for Livno cheeses. This fact led us to a hypothesis that the weight and surface of the cheese also play a role in BA contents, especially in soft smear-ripened cheeses, which are characterised by their shape and microbial diversity [22].

The evaluation of the relationships between BA contents and some characteristics of OT samples (weight, specific surface area, pH, and salt content) revealed interesting findings (Table 4). Correlation coefficients indicated that with increasing cheese weight, BA4 content decreased (r = −0.6374; *p* < 0.001). The highest negative correlation was found for PUT (−0.7375; *p* < 0.001). The relationships between specific surface area and PUT, CAD, TYM, and BA4 contents were positive, the strongest for TYM (+0.3775; *p* < 0.001). For HIM content, no statistically significant correlations were found. 

Unfortunately, pH and salt content were not determined in all samples. Therefore, the correlation coefficients were negligible to minor and nonsignificant. However, a particular trend is evident. Except for HIM, pH values were positively correlated with BA contents, which probably affects the alkaline character of BAs. Positive statistically significant correlations were also found by Komprda et al. [25] for PUT (r = 0.49), TYM (r = 0.44), and CAD (n = 0.41).

Negative correlations between salt content and BA contents were found, the highest for HIM (−0.2609) and BA4 (−0.1328). Increased salt content probably suppresses the activity of microorganisms and thus the development of BA, even though most microorganisms in cheese cultures are resistant to salt content [22]. The effect of the salt content on BA contents was also confirmed by Gentès et al. [44]. The authors found a substantial decrease (up to 38%) in BA contents (PUT, HIM, TYM) at increasing salt content (from 1.2 to 1.7%) in cheddar.

Recently, there has been a general tendency to reduce the salt content of foods, including cheeses [45,46,47]. However, salt is important in technology and also affects cheese quality. Therefore, any reduction in salt content should be preceded by studying the effect on other qualitative parameters of cheese, which may include BA contents.

The BA content of foods is evaluated not only in terms of potential toxicity but also as a quality indicator of the freshness/deterioration rate [3]. For example, the traditional index developed by Mietz and Karmas [48] is used to assess the freshness of fish. This index is based on the increased PUT, CAD, and HIM contents and the decreased SPD and SPM contents during fish storage. Our findings similarly showed a decrease in SPD and SPM, i.e., PAs, and an increase in BAs during the cheese-ripening process. It can be assumed that for ripened cheeses, such an index could help evaluate the degree of ripening and also its safety for sensitive persons. This index would naturally need to consider TYM as the most problematic BA.

## 4. Conclusions

Due to the severity of the effects of biogenic amines, it is essential to accurately assess the aspects under which these compounds form in a specific type of smear-ripened cheese. In our study, the biogenic amine contents were found to vary mainly between batches characterised by cheese shape, month, and year, while the other factors evaluated were less important. Although rings were found to have lower biogenic amine contents than cylinders and small discs, the shape cannot be considered a reliable parameter in decision making. The salt content may also play an important role, as it shows low negative correlations with the content of biogenic amines, the highest in histamine content (−0.2609).

Tyramine was the main biogenic amine in 65% of the batches examined and its content ranged widely from 3.17 to 64.6 mg/kg. Taking into account the highest tyramine content found, 93 g of Olomoucké tvarůžky would represent a critical intake (i.e., 6 mg) for very sensitive persons. Therefore, these cheeses can be considered safe for people without health problems. On the other hand, risk groups, such as patients treated with monoamine oxidase inhibitors, should rather avoid them or consume them in very low quantities. 

## Figures and Tables

**Table 1:** The physical characteristics, number of sampling, and analysed samples of Olomoucké tvarůžky shapes during the observed period (2011–2013).

Shape	Physical Characteristics of One Piece			Number of Samplings	Number of Analysed Samples *		
	Weight (g)	Surface Area (mm^2^)	Specific Surface Area (mm^2^/g)		2011	2013	2019
Rings	29.9 ± 0.9	6645 ± 314	222 ± 9	6	10	10	10
Cylinders	30.6 ± 0.7	5334 ± 436	174 ± 12	4	10	5	5
Discs–small	18.9 ± 0.4	4599 ± 188	243 ± 11	4	5	10	5
Discs–large	27.2 ± 1.2	6082 ± 323	224 ± 12	3	5	10	0
Total	-	-	-	17	30	35	20

* Each sample was analysed as duplicate.

**Table 2:** The parameters of the analytical method.

	PUT	CAD	HIM	TYM	SPD	SPM
LOD (mg/kg)	0.33	0.45	0.75	0.80	0.44	0.46
LOQ (mg/kg)	1.00	1.50	2.50	2.50	1.50	1.50
RSD (%)	3.48	3.21	3.44	4.39	4.57	4.08
Recovery (%)	88	90	87	91	88	85

LOD = limit of detection; LOQ = limit of quantification; RSD = relative standard deviation; PUT = putrescine; CAD = cadaverine; HIM = histamine; TYM = tyramine; SPD = spermidine; SPM = spermine.

**Table 3:** The average contents (mg/kg) of main biogenic amines and polyamines in Olomoucké tvarůžky cheeses according to selected factors.

Batch Shape (Month/Year)		PUT		CAD		HIM		TYM		SPD		SPM		BA4		PA2	
		Mean	SD	Mean	SD	Mean	SD	Mean	SD	Mean	SD	Mean	SD	Mean	SD	Mean	SD
Rings 1 (June/2011)		6.02 ^ab^	2.68	8.79 ^a^	5.69	14.7 ^e^	2.75	18.6 ^ab^	2.50	2.15 ^bc^	0.54	3.79 ^abc^	0.87	48.1 ^ab^	12.0	5.94 ^bcd^	1.32
Rings 2 (November/2011)		1.43 ^a^	0.54	5.63 ^a^	3.64	3.90 ^ab^	1.03	15.5 ^a^	2.87	1.69 ^ab^	0.20	1.81 ^a^	0.25	26.5 ^a^	6.66	3.50 ^ab^	0.39
Rings 3 (March/2013)		2.64 ^a^	1.42	1.66 ^a^	0.36	36.2 ^f^	2.54	5.30 ^a^	2.08	5.20 ^g^	0.82	7.17 ^ef^	1.07	45.8 ^ab^	4.74	12.4 ^fg^	1.02
Rings 4 (May/2013)		2.04 ^a^	0.38	1.89 ^a^	0.52	41.3 ^g^	4.83	13.3 ^a^	10.6	9.76 ^i^	1.39	5.32 ^cde^	0.70	58.6 ^abc^	14.4	15.1 ^hi^	1.84
Rings 5 (April/2019)		1.31 ^a^	0.40	2.82 ^a^	1.10	1.51 ^a^	0.64	33.7 ^bc^	26.2	3.36 ^de^	0.48	3.99 ^abc^	1.40	39.3 ^ab^	27.9	7.35 ^cd^	1.41
Rings 6 (November/2019)		4.19 ^a^	4.12	10.5 ^a^	12.2	15.3 ^e^	5.05	39.1 ^cd^	20.4	1.80 ^ab^	0.23	11.4 ^g^	3.47	69.1 ^bcd^	38.3	13.2 ^gh^	3.49
Cylinders 1 (October/2011)		8.45 ^ab^	2.02	3.51 ^a^	1.17	8.75 ^bc^	1.82	50.8 ^def^	9.69	0.95 ^a^	0.17	2.43 ^ab^	0.53	71.5 ^bcde^	13.8	3.38 ^a^	0.67
Cylinders 2 (November/2011)		21.6 ^c^	4.96	69.6 ^d^	21.3	4.44 ^abc^	0.79	8.85 ^a^	2.90	1.61 ^ab^	0.60	3.24 ^abc^	0.69	104 ^ef^	26.9	4.85 ^abc^	1.04
Cylinders 3 (May/2013)		21.1 ^c^	4.36	35.7 ^c^	8.29	57.1 ^h^	3.29	57.6 ^ef^	4.76	4.23 ^efg^	0.45	6.77 ^def^	0.84	171 ^g^	15.7	11.0 ^fg^	1.06
Cylinders 4 (November/2019)		3.45 ^a^	1.17	7.76 ^a^	1.94	13.9 ^de^	3.78	16.9 ^ab^	3.47	1.57 ^ab^	0.28	14.5 ^h^	3.76	42.1 ^ab^	10.1	16.1 ^i^	3.88
Discs–small 1 (October/2011)		36.8 ^d^	5.03	128 ^e^	27.7	41.2 ^g^	5.22	36.1 ^cd^	4.12	1.16 ^ab^	0.23	3.18 ^abc^	0.50	242 ^h^	33.8	4.35 ^ab^	0.67
Discs–small 2 (March/2013)		21.1 ^c^	3.53	28.5 ^bc^	5.89	4.63 ^abc^	0.59	64.6 ^f^	11.7	3.87 ^def^	0.61	4.49 ^bcd^	0.85	119 ^f^	21.2	8.36 ^de^	1.01
Discs–small 3 (April/2013)		2.39 ^a^	0.38	3.46 ^a^	0.99	54.1 ^h^	5.36	37.0 ^cd^	6.42	4.48 ^fg^	0.55	7.95 ^f^	1.67	97.0 ^def^	9.92	12.4 ^fg^	1.61
Discs–small 4 (November/2019)		21.0 ^c^	15.7	16.5 ^ab^	5.76	17.5 ^e^	3.78	53.0 ^def^	18.3	2.04 ^bc^	0.30	8.43 ^f^	1.20	108 ^f^	41.7	10.5 ^ef^	1.44
Discs–large 1 (November/2011)		11.9 ^b^	6.48	26.3 ^bc^	14.6	9.35 ^cd^	1.84	41.3 ^cde^	9.19	1.30 ^ab^	0.58	2.95 ^ab^	0.86	88.8 ^cdef^	31.1	4.26 ^ab^	1.42
Discs–large 2 (March/2013)		4.16 ^a^	0.79	8.88 ^a^	4.21	3.05 ^a^	0.97	8.64 ^a^	5.04	3.00 ^cd^	0.68	7.87 ^f^	2.32	24.7 ^a^	6.71	10.9 ^efg^	1.86
Discs–large 3 (May/2013)		2.13 ^a^	0.41	2.16 ^a^	0.42	38.9 ^fg^	2.26	3.17 ^a^	0.57	8.13 ^h^	1.66	7.81 ^f^	1.32	46.4 ^ab^	2.37	15.9 ^i^	2.16
The effect of (%; *p*-value)	Year	8 ***		18 ***		30 ***		2 ***		56 ***		50 ***		4 ***		63 ***	
	Batch	75 ***		73 ***		68 ***		75 ***		37 ***		32 ***		83 ***		24 ***	
	Storage	1 **		1 **		0 ns		0 ns		0 *		3 ***		1 ns		3 ***	
R^2^ (%)		84.2		92.2		97.4		78.0		93.5		85.6		87.7		89.2	

^a–i^ = means with different superscripts in the row differ significantly (*p* < 0.05; * *p* < 0.05; ** *p* < 0.01; *** *p* < 0.001); SD = standard deviation; PUT = putrescine; CAD = cadaverine; HIM = histamine; TYM = tyramine; SPD = spermidine; SPM = spermine; BA4 = sum of PUT, CAD, HIM, and TYM; PA2 = sum of SPD and SPM.

**Table 4:** Pearson correlation coefficients between selected characteristics of Olomoucké tvarůžky cheeses and contents (mg/kg) of biogenic amines. Statistic characteristics of weight, specific surface area, pH, and salt content are expressed as mean ± standard deviation and range (min–max).

	Weight (g) (n = 82)	Specific Surface Area (mm^2^/g) (n = 82)	pH (n = 40)	Salt Content (%) (n = 40)
	26.98 ± 4.79 (17.9–32.1)	218 ± 26 (154–272)	7.27 ± 0.68 (6.14–8.31)	4.95 ± 0.22 (4.42–5.46)
PUT	−0.7375 ***	0.3626 ***	0.2130	−0.1152
CAD	−0.5010 ***	0.3204 **	0.0190	−0.0947
HIM	−0.2162	0.1921	−0.1560	−0.2609
TYM	−0.4418 ***	0.3775 ***	0.1778	−0.0692
BA4	−0.6374 ***	0.4349 ***	0.1303	−0.1328

** *p* < 0.01; *** *p* < 0.001; PUT = putrescine; CAD = cadaverine; HIM = histamine; TYM = tyramine; BA4 = sum of PUT, CAD, HIM, and TYM.

## Data Availability

The datasets generated for this study are available on reasonable request to the corresponding author.
